# Supplementary material for: Content-rich biological network constructed by mining PubMed abstracts
Source: BMC Bioinformatics. 2004 Oct 8;5:147. doi: 10.1186/1471-2105-5-147 (PMC528731; doi:10.1186/1471-2105-5-147)
Supplement: Additional File 2 — The original results of the above study (non-essential files are deleted to keep the file size under the limit set by BMC bioinformatics). [file 1471-2105-5-147-S2.bz2 › chilibotAdditionalFile2/dip40/636ID11183778E11023/html/GTF2F1_GTF2B.html]

 


 **GTF2F1** and **GTF2B** 
  
Found 80 abstracts in PubMed, retrieved 40.  
 

 What does Google say? 
 PDF only 
| .edu only 

---

**Interactive relationship** (e.g. stimulation, inhibition, etc)

**Neutral relationship**- It has been shown that cterRAP74 and the core domain of hTFIIB TFIIBc, residues 112 316 directly interact with the carboxyl terminal domain of hFCP1 cterFCP, residues 879 961, and these interactions may be responsible for the regulatory activities of TFIIF  [ **GTF2F1** ]  and TFIIB  [ **GTF2B** ]  on FCP1.  Ref: 12578358 Biochemistry< MedlineTA>Biochemistry,
- Initiation factors, TFIIB  [ **GTF2B** ] , TFIIE beta, and TFIIF  [ **GTF2F1** ]  beta as well as the TATA box binding protein TBP were found to interact with BTEB2 when analyzed by in vitro binding experiments, although these interactions could not be attributed to the proline rich domain.  Ref: 9089417 J Biochem Tokyo , 1997
- According to solution and mutagenesis studies, the multiple domains of RAP30 and RAP74  [ **GTF2F1** ]  bind PolII, TFIIB  [ **GTF2B** ] , TAF250 and DNA in interactions that are essential for transcription initiation and elongation.  Ref: 11183778 J Mol Biol, 2000
- The interaction between RMP and TFIIF  [ **GTF2F1** ]  may be an additional passway for RMP to regulate the transcription, or alternatively TFIIF  [ **GTF2F1** ]  may cooperate with RPB5 and TFIIB  [ **GTF2B** ]  for the corepressor function of RMP.  Ref: 12737519 Cell Res, 2003
- A motif shared by TFIIF  [ **GTF2F1** ]  and TFIIB  [ **GTF2B** ]  mediates their interaction with the RNA polymerase II carboxy terminal domain phosphatase Fcp1p in Saccharomyces cerevisiae.  Ref: 11003641 Mol Cell Biol, 2000
- These regulatory interactions with Fcp1p involved closely related amino acid sequence motifs in TFIIB  [ **GTF2B** ]  and RAP74  [ **GTF2F1** ] .  Ref: 11003641 Mol Cell Biol, 2000
- Interestingly both RAP30 and RAP74  [ **GTF2F1** ]  interact with the same domain D5 of the C terminal RMP of 118 amino acid residuals which overlaps with its TFIIB  [ **GTF2B** ]  binding domain.  Ref: 12737519 Cell Res, 2003
- TFIIB  [ **GTF2B** ]  contains a zinc binding motif near the N terminus that is directly involved in the interaction with RNA pol II TFIIF  [ **GTF2F1** ]  and plays a crucial role in selecting the transcription initiation site.  Ref: 11045620 Protein Sci, 2000
- Using interaction Far Western blotting assays, we systematically tested for protein protein interactions between Msx2 and components of the basal transcriptional machinery known to mediate transcriptional activation TBP, TFIIB  [ **GTF2B** ] , and TFIIF  [ **GTF2F1** ] .  Ref: 9265625 Biochemistry, 1997
- Msx2 binds both components of TFIIF  [ **GTF2F1** ]  RAP74  [ **GTF2F1** ] , RAP30, but not TFIIB  [ **GTF2B** ]  or TBP.  Ref: 9265625 Biochemistry, 1997
- In initiation, TFIIF  [ **GTF2F1** ]  induces promoter DNA to wrap almost a full turn around RNA polymerase II in a complex that includes the general transcription factors TATA binding protein, TFIIB  [ **GTF2B** ] , and TFIIE.  Ref: 10567562 Mol Cell Biol, 1999
- The N terminal domain of RAP74  [ **GTF2F1** ]  minimally aa 1 to 172 is sufficient to deliver pol II into a complex formed on the adenovirus major late promoter with the TATA binding protein, TFIIB  [ **GTF2B** ] , and RAP30.  Ref: 9528785 Mol Cell Biol, 1998
- The phosphatase activity of FCP1 is strongly enhanced by the carboxyl terminal domain of RAP74  [ **GTF2F1** ]  cterRAP74, residues 436 517, and this stimulatory effect of TFIIF  [ **GTF2F1** ]  can be blocked by TFIIB  [ **GTF2B** ] .  Ref: 12578358 Biochemistry< MedlineTA>Biochemistry, 1998
- Stepwise analysis of preinitiation complex formation by electrophoretic mobility shift assay revealed that the phosphorylation of TBP and TFIIB  [ **GTF2B** ]  by DNA PK did not affect the formation of promoter P TBP and P TBP TFIIB  [ **GTF2B** ]  complexes but synergistically stimulated the formation of P TBP TFIIB  [ **GTF2B** ]  TFIIF  [ **GTF2F1** ]  Pol II complex.  Ref: 9288944 Eur J Biochem, 1997
- TFIIB  [ **GTF2B** ]  interacts with a promoter complex containing the TATA binding protein TBP to facilitate interaction with RNA polymerase II RNA pol II and the associated transcription factor IIF TFIIF  [ **GTF2F1** ] .  Ref: 11045620 Protein Sci, 2000

**Non-interactive relationship** (e.g. studied together, co-existance, homology, etc.)

- Our results show that a system consisting of purified recombinant TFIIA, TFIIB  [ **GTF2B** ] , TFIIE, TFIIF  [ **GTF2F1** ] , CREB, and Tax, along with highly purified RNA polymerase II, affinity purified epitope tagged TFIID, and semipurified TFIIH, supports basal transcription of the HTLV 1 promoter but is not responsive to Tax.  Ref: 10666246 J Virol, 2000
- Interestingly, preincubation of TATA binding protein TBP or TFIID with HPV template is not sufficient to overcome E2 mediated repression, which can be alleviated only via formation of a minimal TBP or TFIID TFIIB  [ **GTF2B** ]  RNA polymerase II TFIIF  [ **GTF2F1** ]  preinitiation complex.  Ref: 10594014 Mol Cell Biol, 2000
- The measurements showed comparable amounts of RNA polymerase II, TFIIE, and TFIIF  [ **GTF2F1** ] , lower levels of TBP and TFIIB  [ **GTF2B** ] , and still lower levels of Mediator and TFIIH.  Ref: 11591727 J Biol Chem, 2001
- With the availability of the general initiation factors TFIIB  [ **GTF2B** ] , TFIID, TFIIE, TFIIF  [ **GTF2F1** ] , and TFIIH, it is now possible to investigate aspects of the mechanism of eukaryotic messenger RNA synthesis in purified, reconstituted RNA polymerase II transcription systems.  Ref: 9237163 Methods, 1997
- Here we describe cloning of the mouse cDNAs encoding TFIIB  [ **GTF2B** ]  and the small and large TFIIE and TFIIF  [ **GTF2F1** ]  subunits.  Ref: 11502214 Eur J Biochem, 2001
- The zinc ribbon structure of TFIIB  [ **GTF2B** ] , in conjunction with the biochemical analyses, suggests that residues on the beta sheet are involved in the interaction with RNA pol II TFIIF  [ **GTF2F1** ] , while the zinc binding site may increase the stability of the beta sheet.  Ref: 11045620 Protein Sci, 2000
- A mouse in vitro transcription system reconstituted from highly purified RNA polymerase II, TFIIH and recombinant TBP, TFIIB  [ **GTF2B** ] , TFIIE and TFIIF  [ **GTF2F1** ] .  Ref: 11502214 Eur J Biochem, 2001
- Furthermore, we found that the recruitment of TFIIF  [ **GTF2F1** ]  and RNA polymerase II to a TFIID TFIIA TFIIB  [ **GTF2B** ]  promoter complex dictates the rate of preinitiation complex formation.  Ref: 11743717 J Mol Biol, 2001
- Here we report that Gal11p stimulates basal transcription in a reconstituted transcription system composed of recombinant or highly purified transcription factors, TFIIB  [ **GTF2B** ] , TFIIE, TFIIF  [ **GTF2F1** ] , TFIIH, and TATA box binding protein and core RNA polymerase II.  Ref: 8790357 Proc Natl Acad Sci U S A, 1996
- Co expression of RAP74  [ **GTF2F1** ]  in MC3T3 E1 cells partially reverses >50% suppression of OC promoter activity by Msx2, while co expression of TFIIB  [ **GTF2B** ]  or RAP30 has no effect.  Ref: 9265625 Biochemistry, 1997
- Human recombinant TATA binding protein, transcription factor TF IIB  [ **GTF2B** ]  and TFIIF  [ **GTF2F1** ]  made up of the 30 and 74 kDa RNA polymerase II associated proteins RAP30 and RAP74  [ **GTF2F1** ]  were incubated with calf thymus poly ADP ribose polymerase and 32P NAD at 37 degrees C.  Ref: 9164864 Biochem J, 1997
- To address this, we used formaldehyde crosslinked chromatin immunoprecipitation X ChIP to map TATA binding protein TBP, transcription initiation factor IIB  [ **GTF2B** ]  TFIIB  [ **GTF2B** ]  and IIF TFIIF  [ **GTF2F1** ] , and dHDAC1 RPD3 across several Drosophila promoter regions.  Ref: 11493924 Nature, 2001
- Interestingly, multiple basal transcription factors for RNA polymerase II, including TBP, TFIIB  [ **GTF2B** ] , TFIIH, and TFIIF  [ **GTF2F1** ] , were found to be associated with MSS1.  Ref: 11118327 Biochem Biophys Res Commun, 2000
- About 18 00 molecules of polymerases approximately 11 00 molecules of form IIO and 1 00 to 3 00 molecules of each of TFIIB  [ **GTF2B** ] , TFIIEalpha, TFIIEbeta, TFIIF  [ **GTF2F1** ]  RAP74  [ **GTF2F1** ] , TFIIF  [ **GTF2F1** ]  RAP30, and TFIIH MAT1 remained tightly associated with the nuclear substructure.  Ref: 10409729 Mol Cell Biol, 1999
- These results provide new information about the recognition of acidic rich activation domains involved in transcriptional regulation, and provide insights into how TFIIF  [ **GTF2F1** ]  and TFIIB  [ **GTF2B** ]  regulate the FCP1 phosphatase activity in vivo.  Ref: 12578358 Biochemistry< MedlineTA>Biochemistry, 1999
- The core promoter is the site for assembly of the transcription preinitiation complex, which includes RNA pol II and the general transcription fctors TBP, TFIIB  [ **GTF2B** ] , TFIIE, TFIIF  [ **GTF2F1** ] , and TFIIH.  Ref: 9618449 Microbiol Mol Biol Rev, 1998
- The Cdc73p Paf1p RNA polymerase II containing complex also includes Gal11p, and the general initiation factors TFIIB  [ **GTF2B** ]  and TFIIF  [ **GTF2F1** ] , but lacks TBP, TFIIH, and transcription elongation factor TFIIS as well as the Srbps.  Ref: 9032243 Mol Cell Biol, 1997
- A complex containing TBP, TFIIB  [ **GTF2B** ] , TFIIF  [ **GTF2F1** ] , and RNAPII is capable of forming on the promoter in an Inr dependent manner.  Ref: 9154795 Mol Cell Biol, 1997
- Solution structure of the carboxyl terminal domain of RAP74  [ **GTF2F1** ]  and NMR characterization of the FCP1 binding sites of RAP74  [ **GTF2F1** ]  and human TFIIB  [ **GTF2B** ] .  Ref: 12578358 Biochemistry< MedlineTA>Biochemistry, 1997
- Instead, general initiation factors transcription factor TF IIB  [ **GTF2B** ] , TFIID, TFIIE, TFIIF  [ **GTF2F1** ] , and TFIIH assemble on promoter DNA with polymerase II, creating a large multiprotein DNA complex that supports accurate initiation.  Ref: 8990153 Proc Natl Acad Sci U S A, 1997
- The DNA wrapping model describes specific roles for general RNA polymerase II transcription factors TATA binding protein TBP, TFIIB  [ **GTF2B** ] , TFIIF  [ **GTF2F1** ] , TFIIE, and TFIIH, provides a plausible explanation for preinitiation complex isomerization, suggests mechanisms underlying the synergy between transcriptional activators, and suggests an unforseen role for TBP associating factors in transcription.  Ref: 10357858 Microbiol Mol Biol Rev, 1999
- TATA binding protein TBP, transcription factor TF IIB  [ **GTF2B** ] , TFIIE, TFIIF  [ **GTF2F1** ] , and TFIIH.  Ref: 11502214 Eur J Biochem, 2001
- Two portions of this carboxy terminal region of Fcp1p bound directly to the first cyclin like repeat in the core domain of the general transcription factor TFIIB  [ **GTF2B** ] , as well as to the RAP74  [ **GTF2F1** ]  subunit of TFIIF  [ **GTF2F1** ] .  Ref: 11003641 Mol Cell Biol, 2000
- The eluted RAP fraction did not contain the holoenzyme components Srb, 5 6p, Gal11p, or Sug1p, but did include the known transcription factors TFIIB  [ **GTF2B** ]  and TFIIS and the three subunits of yeast TFIIF  [ **GTF2F1** ]  Ssu71p Tfg1p, Tfg2p, and Anc1p Tfg3p.  Ref: 8812838 Protein Expr Purif, 1996
- Our results indicate that hATF4 interacts with several general transcription factors TATA binding protein, TFIIB  [ **GTF2B** ] , and the RAP30 subunit of TFIIF  [ **GTF2F1** ] .  Ref: 9295363 J Biol Chem, 1997
- In this study, we noted that the extent of activation by ICP4 in vitro was highly dependent on the purity of TFIID when recombinant TFIIB  [ **GTF2B** ] , TFIIE, and TFIIF  [ **GTF2F1** ]  were used as sources of these factors.  Ref: 9658123 J Virol, 1998
- Using order of addition and template challenge assays performed in a human cell free transcription system reconstituted with recombinant general transcription factors TFIIB  [ **GTF2B** ] , TBP, TFIIE, TFIIF  [ **GTF2F1** ] , a recombinant general cofactor PC4, and highly purified epitope tagged multiprotein complexes TFIID, TFIIH, pol II, we demonstrate that when TBP is used as the TATA binding factor transcriptional activators such as Gal4 VP16 and human papillomavirus E2 mainly function by facilitating pol II entry to the promoter region.  Ref: 11457828 J Biol Chem, 2001
- Alleviation of human papillomavirus E2 mediated transcriptional repression via formation of a TATA binding protein or TFIID TFIIB  [ **GTF2B** ]  RNA polymerase II TFIIF  [ **GTF2F1** ]  preinitiation complex.  Ref: 10594014 Mol Cell Biol, 2000
- This holoenzyme contained nearstoichiometric amounts of all the general transcription factors, TFIIB  [ **GTF2B** ] , TFIID TBP TAFIIs, TFIIE, TFIIF  [ **GTF2F1** ] , and TFIIH, required to accurately initiate transcription in vitro at the adenovirus major late promoter.  Ref: 9305922 J Biol Chem, 1997
- By exploiting adenovirus 2 major late promoter derivatives that contain premelted transcriptional start sites and do not require TFIIF  [ **GTF2F1** ] , TFIIE, or TFIIH for transcription initiation, we observe i that Elongin is capable of strongly stimulating the rate of synthesis of trinucleotide transcripts by a subcomplex of RNA polymerase II, TBP, and TFIIB  [ **GTF2B** ]  and ii that the ability of Elongin to stimulate synthesis of these short transcripts is substantially reduced by addition of TFIIF  [ **GTF2F1** ]  to transcription reactions.  Ref: 9756900 J Biol Chem, 1998
- Finally, when using TBP mutants defective on multiple interaction surfaces, we observed synthetic lethal effects between mutations on the TFIIA and TFIIB  [ **GTF2B** ]  interfaces but found that mutations implicated in association with polymerase II and TFIIF  [ **GTF2F1** ]  did not have significant effects in vivo.  Ref: 9032260 Mol Cell Biol, 1997
- This model stipulates that wrapping of the promoter DNA around RNAPII and the general initiation factors TBP, TFIIB  [ **GTF2B** ] , TFIIE, TFIIF  [ **GTF2F1** ]  and TFIIH induces a torsional strain in the DNA double helix that facilitates strand separation and open complex formation.  Ref: 11509574 J Biol Chem, 2001
- These lines of evidence suggest that TIP120 is able to activate basal transcription by overcoming a kinetic impediment to RNAP II TFIIF  [ **GTF2F1** ]  integration into the TBP TFIID TFIIB  [ **GTF2B** ]  DNA complex.  Ref: 10567521 Mol Cell Biol, 1999
